# Magnetic Field Effect on the Electric and Dielectric Properties of the Linear Magnetoelectric Compound Co_4_Nb_2_O_9_

**DOI:** 10.3390/ma17235719

**Published:** 2024-11-22

**Authors:** Iliana N. Apostolova, Angel T. Apostolov, Julia M. Wesselinowa

**Affiliations:** 1 Faculty of Forest Industry, University of Forestry, 1756 Sofia, Bulgaria; inaapos@abv.bg; 2 Department of Physics, Faculty of Hydrotechnics, University of Architecture, Civil Engineering and Geodesy, 1046 Sofia, Bulgaria; angelapos@abv.bg; 3 Faculty of Physics, Sofia University “St. Kliment Ohridski”, J. Bouchier Blvd. 5, 1164 Sofia, Bulgaria

**Keywords:** Co4Nb2O9, magnetization, polarization, dielectric constant, microscopic model, Green’s function theory

## Abstract

Using Green’s function theory and a microscopic model, the multiferroic properties of ${\mathrm{Co}}_{4}{\mathrm{Nb}}_{2}O_{9}$ are investigated theoretically. There are some discrepancies in the discussion of the electric and dielectric behavior of CNO with and without external magnetic fields. We try to clarify them. It is observed that the polarization and the dielectric constant do not show a peak at the antiferromagnetic phase transition temperature $T_{N}$ without an external magnetic field *h*. But applying *h*, there appears a peak around the Neel temperature $T_{N}$, which increases with increasing *h* and then shifts to lower temperatures. The magneto-dielectric coefficient MD(T,h) is also calculated. Moreover, the magnetization rises with an increasing external electric field below the Neel temperature. This shows strong magnetoelectric coupling in ${\mathrm{Co}}_{4}{\mathrm{Nb}}_{2}O_{9}$. The obtained results are compared with the existing experimental data. There is a good qualitative agreement.

## 1. Introduction

Magnetoelectric (ME) materials, which exhibit both magnetism and electric polarization within a single phase, have given rise to significant interest due to their unique physical properties and diverse applications [1,2]. In ME materials, it is possible to control the magnetization using an external electric field and, conversely, control the electric polarization with a magnetic field. Khomskii [3] introduced a classification of multiferroics into two categories: type-I and type-II. In type-I multiferroics, such as hexagonal ${\mathrm{RMnO}}_{3}$ [4], the critical temperatures for ferroelectric $T_{C}$ and magnetic $T_{N}$ transitions are distinctly separated, often well above room temperature, with $T_{C}$ being much higher than $T_{N}$. The ME coupling in these materials is typically quadratic and relatively weak. In contrast, type-II multiferroics, like orthorhombic ${\mathrm{RMnO}}_{3}$ [5], exhibit magnetic ordering that breaks the inversion symmetry, which directly leads to ferroelectricity. In magnetically driven multiferroics, long-range magnetic order, which lacks centrosymmetry, induces macroscopic electric polarization. The transition temperatures for both magnetic and ferroelectric phases are nearly identical, and the ME coupling tends to be linear and significantly stronger.

Another example of materials in the second class, where the ME coupling is linear, exhibit electric polarization only when external magnetic fields are applied. This class includes materials such as ${\mathrm{MnTiO}}_{3}$ [6], ${\mathrm{NdCrTiO}}_{5}$ [7], and ${\mathrm{Co}}_{4}{\mathrm{Nb}}_{2}O_{9}$ (CNO) [8]. CNO has been found to crystallize in the P$\bar{3}$c1 space group, associated with the α${\mathrm{-Al}}_{2}O_{3}$ structure, and shares structural similarity with linear ME oxides like ${\mathrm{Fe}}_{4}{\mathrm{Nb}}_{2}O_{9}$ and ${\mathrm{Mn}}_{4}{\mathrm{Nb}}_{2}O_{9}$. CNO is an antiferromagnetic material within the $M_{4}{\mathrm{M’}}_{2}O_{9}$ family (M = Mn, Fe, and Co; M’ = Nb and Ta). In this material, dielectric anomalies and electric polarization appear near the antiferromagnetic transition temperature $T_{N}$, but only when applying an external magnetic field [9]. Recent findings have shown that CNO possesses a high ME coupling coefficient [9,10]. The significant ME and magneto-dielectric effects observed in CNO have been linked to a spin-flop phase transition induced by magnetic interactions. The Neel temperature of this material is $T_{N}$≈ 28 K [9,10]. The first study reporting the effects of an external electric field on CNO’s magnetic properties was conducted by Fischer et al. [8]. In CNO, Co ions occupy two distinct sites, although there is some controversy regarding the exact nature of the magnetic configuration. Earlier research by Bertaut et al. [11] suggested a linear magnetic arrangement, where the magnetic moments of two ${\mathrm{Co}}^{2+}$ ions are antiparallel and aligned along the crystallographic *c*-axis. Khanh et al. [12] demonstrated that the magnetization and ME properties of CNO do not align with a purely linear magnetic structure, proposing instead that the magnetic moments are canted within the ab plane at an angle relative to the *c*-axis. Srivastava et al. [13] provided evidence of spontaneous electric polarization in CNO, even without an external magnetic field below $T_{N}$. Neutron powder diffraction studies by Deng et al. [14] revealed that CNO adopts a noncollinear magnetic structure, with ${\mathrm{Co}}^{2+}$ moments oriented in the ab plane. This canted spin arrangement is identified as the source of the material’s strong ME coupling. It should be noted that CNO is not a highly frustrated system magnetically, and the emergence of a canting effect in the ab-plane is a result of the high value of the Dzyaloshinskii–Moriya interaction (DMI) [14]. A dynamic model was proposed to account for the observed spin dynamics. Theoretical investigations into linear ME coupling and the modulation of electric polarization by external magnetic fields in CNO have been conducted using the orbital model [15], symmetry analysis [16], and Hartree–Fock calculations [17].

In this paper we propose a microscopic model and Green’s function theory in order to explain theoretically the multiferroic properties of CNO and to clarify the discrepancies in the discussion of the electric and dielectric behavior of CNO with and without external magnetic fields. Is it an antiferromagnetic and antiferroelectric compound? The results obtained are compared with existing experimental data and are in good qualitative agreement with the most of them.

## 2. Model and Method

CNO exhibits antiferromagnetic behavior below $T_{N}$, with a spin-flop transition occurring at a critical field *h*. Based on the magnetic structure outlined by Deng et al. [14], the Hamiltonian that characterizes the magnetic properties of CNO is expressed as follows:

(1) $$\begin{array}{ccc} H_{m} & = & -\sum_{ij}J_{1ij}S_{i}^{1}\cdot S_{j}^{2}-\sum_{ij}J_{2ij}S_{i}^{1}\cdot S_{j}^{2}-\sum_{ij}J_{3ij}S_{i}^{1}\cdot S_{j}^{2}\\ & - & \sum_{i}K_{1i}{\left(S_{i}^{z1}\right)}^{2}-\sum_{i}K_{2i}{\left(S_{i}^{z2}\right)}^{2}-\sum_{ij}D_{ij}\cdot \left(S_{i}^{2}\times S_{j}^{2}\right)\\ & - & g\mu_{B}h\cdot \sum_{i}S_{i}^{1,2}. \end{array}$$

**Figure 1:**
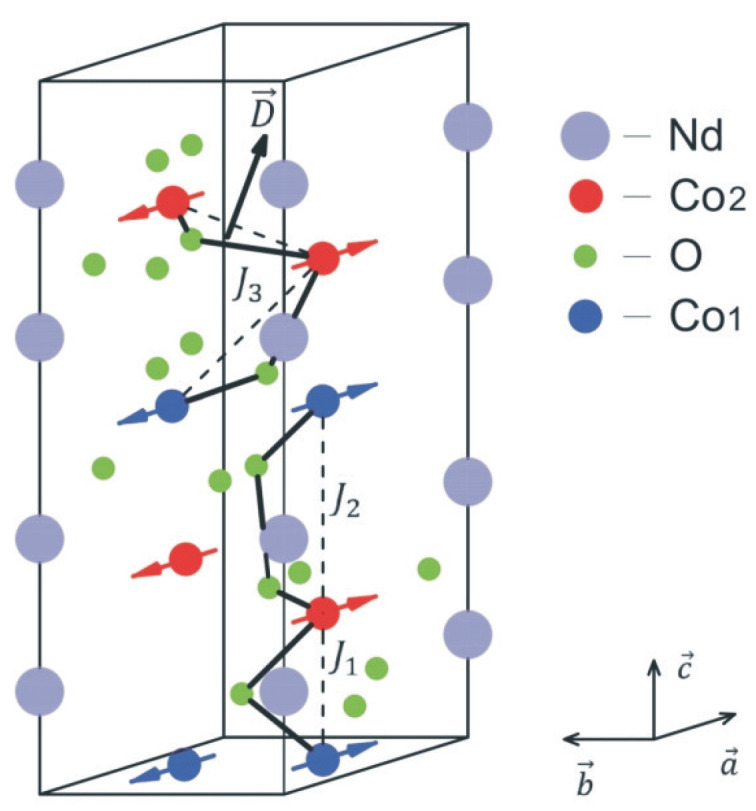
(Color online) The crystalline and magnetic structure of CNO.

The operators $S_{i}^{1,2}$ represent the Heisenberg spin operators for the Co1 and Co2 sites. $J_{1ij}$ and $J_{2ij}$ correspond to the ferromagnetic super-exchange interactions between the nearest (Co1-O-Co2) and next-nearest (Co1-O-O-Co2) Co ions along the *c*-axis, while $J_{3ij}$ describes the antiferromagnetic exchange interaction within the ab-plane between Co1-O-Co2. We have neglected the super-exchange interaction between any two neighbor Co1 ions in the planar network Co1-O-Co1 (which should be ferromagnetic), assuming that this interaction might be very weak following the experimental data of Deng et al. [14]. $D_{ij}$ is the DMI between two neighboring ${\mathrm{Co}}^{2+}$ ions in the ab-plane, which causes the noncollinear in-plane magnetic structure of CNO (see Figure 1). $K_{1}$ and $K_{2}$ represent the easy-plane anisotropies for the Co1 and Co2 ions, respectively. It should be emphasized that the single-ion anisotropy *K* in CNO is significantly larger than the exchange interactions. *h* is an external magnetic field.

The magnetization is observed from

(2) $$M=|M^{1}+M^{2}|$$

with

(3) $$M^{1,2}=\langle S^{z1,2}\rangle =\frac{1}{N}\sum_{ij}\left[\left(S+0.5\right)\coth\left[\left(S+0.5\right)\beta E_{ij}^{1,2}\right)]-0.5\coth\left(0.5\beta E_{ij}^{1,2}\right)\right].$$

In this context, *S* denotes the spin quantum number, $\beta =1/k_{B}T$ is the inverse thermal energy, and $E_{ij}$ represents the spin excitations obtained from the spin Green’s function $G_{ij}=\ll S_{i}^{+};S_{j}^{-}\gg$, calculated using the approach developed by Tserkovnikov [18]. After performing a formal integration of the equation of motion for the retarded two-time Green’s function, the solution can be determined.

(4) $$G_{ij}\left(t\right)=\langle \langle S_{i}^{+}\left(t\right);S_{j}^{-}\rangle \rangle$$

one obtains

(5) $$G_{ij}\left(t\right)=-i\theta \left(t\right)\langle \left[S_{i}^{+};S_{j}^{-}\right]\rangle \exp\left(-iE_{ij}\left(t\right)t\right),$$

with θ(t) = 1 for t>0 and θ(t) = 0 for t<0,

(6) $$\begin{array}{ccc} E_{ij}\left(t\right)=E_{ij} & - & \frac{i}{t}\int_{0}^{t}dt^{\prime }t^{\prime }\left(\frac{\langle \left[j_{i}\left(t\right);j_{j}^{+}\left(t^{\prime }\right)\right]\rangle }{\langle \left[S_{i}^{+}\left(t\right);S_{j}^{-}\left(t^{\prime }\right)\right]\rangle }\right.\\ & - & \left.\frac{\langle \left[j_{i}\left(t\right);S_{j}^{-}\left(t^{\prime }\right)\right]\rangle \langle \left[S_{i}^{+}\left(t\right);j_{j}^{+}\left(t^{\prime }\right)\right]\rangle }{{\langle \left[S_{i}^{+}\left(t\right);S_{j}^{-}\left(t^{\prime }\right)\right]\rangle }^{2}}\right). \end{array}$$

Here, $j_{i}\left(t\right)=\langle \left[S_{i}^{+}\left(t\right),H_{interaction}\right]\rangle$. $E_{ij}$ is the excitation energy beyond the random phase approximation taking into account all correlation functions:

(7) $$E_{ij}=\frac{\langle \left[\left[S_{i}^{+},H\right];S_{j}^{-}\right]\rangle }{\langle \left[S_{i}^{+};S_{j}^{-}\right]\rangle }$$

From the time-dependent term in Equation (6), the damping can be calculated.

It should be noted that another theoretical method is density functional theory (DFT), which is also a very powerful tool in investigating many body problems. However, DFT is mostly concerned with ground-state properties at zero temperature, whereas we are able to make a finite temperature analysis of the excitation spectrum and of all physical quantities. Whereas within DFT all parameters of the system can be—at least in principle—calculated, we are forced to use additional models to find out those parameters. We are convinced that both approaches, DFT and Green’s function method, are well suited and to a certain extent alternatives in describing many body systems.

The magnetically driven polarization is observed from

(8) $$P=Ae_{ij}\times \left(S_{i}\times S_{j}\right).$$

$e_{ij}$ is a unit vector from $S_{i}$ to $S_{j}$, which lies in the (110) plane. *A* is a proportional constant as determined by the spin exchange interaction and the spin–orbit interaction [19,20]. As mentioned in the Introduction, the appearance of polarization P in the system is due to the formation of a canted system in the ab-plane, and to the fact that P is spin-driven. This necessitates the inclusion of the antisymmetric DMI and the associated inverse DM mechanism in the development of the polarization. At zero magnetic field, the presence of two local polarizations that are antiparallel to each other and of equal magnitude was established by Fang et al. [10], which leads to the absence of macroscopic polarization (i.e., in addition to being antiferromagnetic, the compound is also antiferroelectric).

The magnetoelectric term that couples the magnetic and electric subsystems varies linearly with the respective order parameters [14]. We will show that for CNO, the spontaneous electric polarization P is zero without a magnetic field *h*. But applying an external magnetic field *h* induces finite electric polarization, which increases with increasing field *h*, indicating a linear ME effect.

The dielectric constant ϵ(E) is observed from equation [21]:

(9) $$\left({\left(\Lambda /\left(\epsilon \left(E\right)-1\right)\right)}_{\alpha \beta }+\Lambda \right)G^{\alpha \beta }\left(E\right)=\delta_{\alpha \gamma };\;\;\Lambda =4\pi Z^{2}/v,$$

where *Z* is the electron charge; *v*—the volume.

To study the dielectric function ϵ(k,E), we have evaluated the Green’s function $\tilde{G}$:

(10) $$\tilde{G}\left(k,E\right)=\frac{E^{2}-{\left(E_{f}\left(k\right)\right)}^{2}+2iE\gamma^{11}}{\left(E^{2}-{\left(E_{f}\left(k\right)\right)}^{2}+2iE\gamma^{11}\right)\left(E+i\gamma^{33}\right)-E{\left(\epsilon^{13}\right)}^{2}}.$$

$E_{f}\left(k\right)$ and $\gamma^{11}$ are the transverse ferroelectric energy and its associated damping, respectively, while $\gamma^{33}$—the longitudinal damping; the parameter $\epsilon^{13}$—the coupling between the longitudinal and transverse modes. To determine $\epsilon^{\prime }$ and $\epsilon^{\prime \prime }$, we have to analyze the real and imaginary components of the Green’s function (10). From Equation (9), the dielectric function ϵ(k,E) is then observed and investigated for different parameters.

The magneto-dielectric coefficient MD(%) is defined as

(11) MD(%)=[ϵ(h)−ϵ(h=0)]/ϵ(h=0)%.

## 3. Numerical Results and Discussion

For the numerical calculations, which are made with programs in the computer language JAVA, the following model parameters are used: $J_{1}$ = 0.70 meV [14], $J_{2}$ = 0.15 meV [14], $J_{3}$ = −0.52 meV [14], $K_{1}$ = $K_{2}$ = −1.8 meV [14], *D* = 0.22 meV [14], and *A* = $0.12\;{\mathrm{C/m}}^{2}$ [20].

### 3.1. Temperature and Electric Field Dependence of the Magnetization in CNO

**Figure 2:**
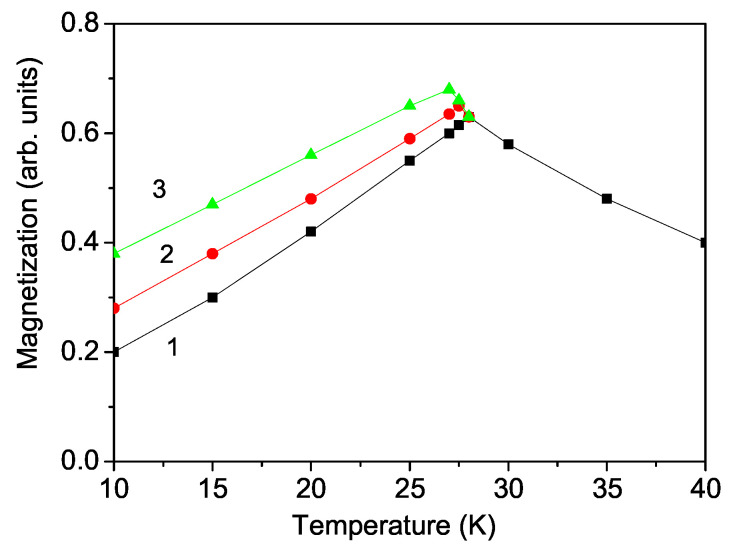
(Color online) Theoretically obtained temperature dependence of the magnetization *M* in CNO for *h* = 1 kOe and different external electric fields *E*: (1) 0, (2) 1, and (3) 2 MV/m.

From Equation (2), we have calculated the temperature dependence of the magnetization M(T) in CNO. The result is presented in Figure 2, curve 1. It can be seen that the magnetization *M* increases below the Neel temperature $T_{N}$∼ 28 K and then decreases above $T_{N}$ with enhancing temperature *T*. This is a typical behavior of an antiferromagnetic compound. Let us emphasize that Fang et al. [10], Cao et al. [22], and Kolodiazhnyi et al. [9] also reported a value of $T_{N}$≈ 28 K, whereas Yadav et al. [23] reported a somewhat smaller value of 27.2 K for CNO. For comparison, we will also point out the values for ${\mathrm{MnTiO}}_{3}$—$T_{N}$ = 64 K [6]—and for ${\mathrm{Cr}}_{2}O_{3}$—$T_{N}$ = 302 K [24]. Unfortunately, Deng et al. [14], who have presented a magnetic structure model, have not calculated analytically and numerically the magnetization.

We have also studied the influence of an external electric field *E* on *M*. It is observed that the magnetization *M* increases with increasing *E* below $T_{N}$, see Figure 2, curves 2 and 3. This is evidence that in CNO there exists strong ME coupling below $T_{N}$. The increase in the magnetization *M* with enhancing *E* can be explained as follows: Without electrical and magnetic fields, there does not exist polarization or magnetization, because the compound is antiferromagnetic and antiferroelectric. But applying an external electric field *E*, there appears a small polarization. Some of the ferroelectric spins are rotated in the *E* direction so that the polarization *P* is now nonzero. Due to the strong ME coupling, there appears a small magnetization *M*, which increases with increasing electric field *E* as well as the electric polarization *P*. The ME coupling is determined by the sensitivity of the change in polarization to the magnetic field and inversely of the magnetization to the electric field. As can be seen from Figure 2 and third picture in Section 3.2, we obtain a strong change in P(h) and in M(E), which is evidence of strong ME coupling. Fang et al. [10] observed from the ME susceptibility P(h) a value of the linear ME coupling α that reaches 18.4 ps/m at 70 kOe. We give for comparison some ME coupling values for other reported linear ME materials, such as ${\mathrm{MnTiO}}_{3}$ (2.6 ps/m) [6] and ${\mathrm{NdCrTiO}}_{5}$ (0.51 ps/m) [7], indicating a large ME coupling in CNO. It must be noted that ${\mathrm{Cr}}_{2}O_{3}$, which shows the same crystal and magnetic structures as CNO, as well the spin-flop-induced electric polarization and cross-coupling between polarization and magnetization, has an ME coupling constant of α = 24.3 ps/m [24]. Above the Neel temperature $T_{N}$, the polarization *P* vanishes and there is no ME coupling between the polarization *P* and the magnetization *M*. Therefore, above $T_{N}$ the electric field *E* cannot tune the magnetization *M*. It can be seen from Figure 2, curves 2 and 3, that the Neel temperature $T_{N}$ decreases slightly with increasing electric field *E*. This behavior is in good qualitative coincidence with the experimental results of Fang et al. [10] for CNO.

### 3.2. Temperature and Magnetic Field Dependence of the Polarization in CNO

**Figure 3:**
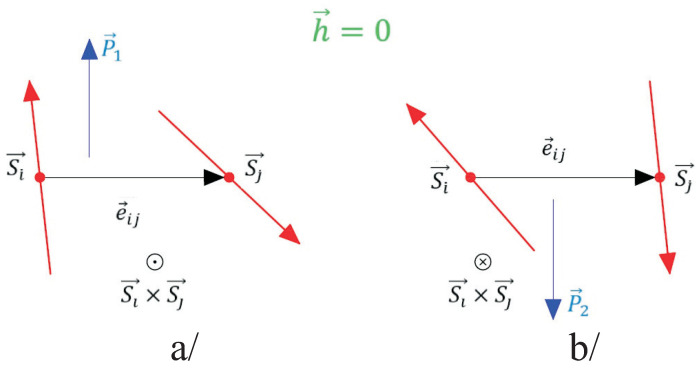
(Color online) Schematic representation of the two sublattice polarizations $P_{1}$ (**a**) and $P_{2}$ (**b**) in the absence of an external magnetic field.

The other physical quantity which is investigated is the polarization *P*. As noted above, the compound CNO is antiferroelectric. In the absence of a magnetic field *h*, there are two sublattices with polarizations in the ab-plane oriented in opposite directions (see Figure 3). The calculation of the polarization in the two sublattices $P_{1}$ and $P_{2}$ is performed using Equation (8), where the total polarization P is expressed as

(12) $$P=P_{1}+P_{2}.$$

**Figure 4:**
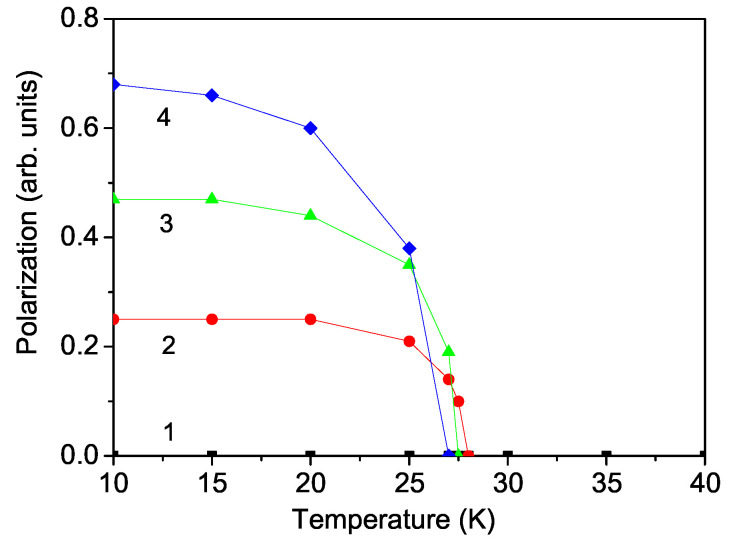
(Color online) Theoretically obtained temperature dependence of the polarization *P* in CNO for different external magnetic fields *h*: (1) 0, (2) 2, (3) 5, and (4) 8 kOe.

The temperature dependence of P for different magnetic field values *h* in the ab-plane is demonstrated in Figure 4. In the case without an external magnetic field *h*, i.e., *h* = 0, the polarization is zero. We have an antiferroelectric behavior (see Figure 4, curve 1), due to the antiparallel spin configuration. This is in agreement with the experimental data for CNO of Deng et al. [14], Khanh et al. [12], and Fang et al. [10] for zero polarization at zero field.

Applying an in-plane magnetic field, the magnetic moments which also lie in the plane rotate slightly to reduce their angles to the external field direction. Due to the ME coupling, some of the ferroelectric spins order in the direction of the magnetic field. Therefore, the total polarization will be nonzero. With an increasing magnetic field, P increases because all ferroelectric spins now point in the same directions. This is caused by the spin-flop transition and there appears an enhanced polarization P (see Figure 4, curves 2–4). The polarization P vanishes at the magnetic phase transition temperature $T_{N}$, which coincides with the ferroelectric phase transition temperature $T_{C}$. The observed tuning of P with the magnetic field *h* is due to the ME coupling and is also evidence that CNO is a linear ME material. Let us emphasize that there is a difference between a multiferroic and a linear ME compound. In both cases, the polarization P depends on the strength and orientation of the magnetic field *h*. The distinction between the two cases is the following. A multiferroic material exhibits ferroelectricity even in the absence of an external magnetic field *h*, whereas electric polarization P in linear ME compounds appears only in the presence of *h*. The observed behavior P(h) shown in Figure 4 is in good qualitative agreement with the experimental data of Fang et al. [10] in CNO and Mufti et al. [6] in ${\mathrm{MnTiO}}_{3}$. The ME measurement by Khanh et al. [12] indicates the ME effect along the *c* axis. This is contradictive to the results of Cao et al. [22] and of Deng et al. [14], which could be due to the slight misalignment of the sample in the measurement.

**Figure 5:**
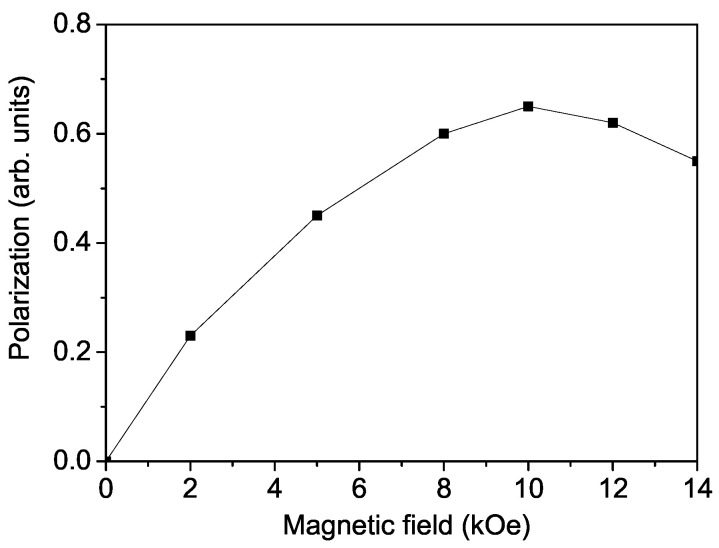
Theoretically obtained magnetic field dependence of the polarization *P* for *T* = 20 K.

**Figure 6:**
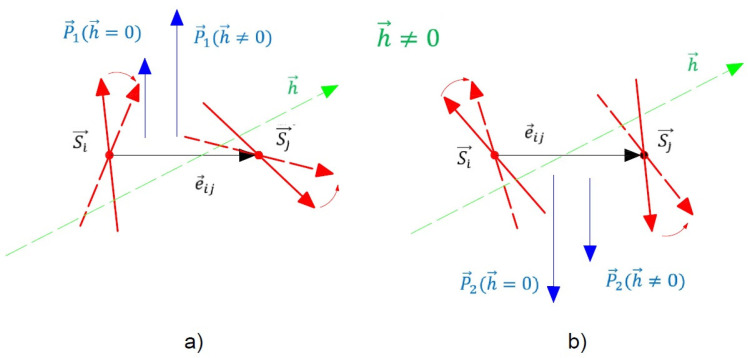
(Color online) Schematic representation of the two sublattice polarizations $P_{1}$ (**a**) and $P_{2}$ (**b**) by applying an external magnetic field in the ab-plane.

In Figure 5 is shown the dependence of P along the [110] direction on the magnetic field *h*, which is applied in the ab-plane. It is clear that as the value of the external magnetic field *h* increases, the polarization P also increases, reaching a maximum value and subsequently decreasing gradually. The qualitative considerations for the behavior of P according to our calculations are as follows. When the angle $\theta_{1}$, which is the angle between neighboring spins $S_{i}$ and $S_{j}$ in the sublattice of $P_{1}$ (see Figure 6a), begins to decrease, approaching $90^{\circ }$, this leads to an increase in $P_{1}$ with an increasing external magnetic field. Conversely, $P_{2}$ will decrease because the angle $\theta_{2}$, which is the angle between the spins $S_{i}^{\prime }$ and $S_{j}^{\prime }$ in the sublattice of $P_{2}$ (see Figure 6b), will increase approaching $180^{\circ }$. This will result in $|P_{1}|>|P_{2}|$, leading to a nonzero total polarization. As the magnetic field increases, the difference between $P_{1}$ and $P_{2}$ will cause P to grow. At a certain critical value of the external magnetic field $h_{cr1}$, $P_{2}$ will become zero because $\theta_{2}=180^{\circ }$, and the spins in Figure 6b will be antiparallel, resulting in $P=P_{1}$. For $h>h_{cr1}$ and $\theta_{2}<180^{\circ }$, $P_{2}$ will become collinear with $P_{1}$, meaning that we will observe a switching of $P_{2}$ by $180^{\circ }$. In this case, $P=P_{1}+P_{2}$, and the total polarization will continue to increase with increasing *h*. Further, an increase in the magnetic field at $h=h_{cr2}$, will lead to a maximum value of P, after which P will begin to decrease. That is, the values of both $\theta_{1}$ and $\theta_{2}$ will become smaller than $90^{\circ }$, and with a further increase in *h*, both $P_{1}$ and $P_{2}$ will start to decrease. For sufficiently high values of the external magnetic field, both $\theta_{1}$ and $\theta_{2}$ will sequentially become zero, meaning the spins in the ab-plane will align in the direction of *h*, becoming collinear, and consequently both $P_{1}$ and $P_{2}$ will become zero, as expressed by Equation (8), i.e., no spontaneous polarization P will be observed. In our opinion, these high values of the external magnetic field have not been experimentally reached due to the large magnetocrystalline anisotropy of the Co ions, which implies a large value for the external magnetic field. From a practical perspective, we are primarily interested in achieving the maximum value of spin-induced polarization when applying *h* in the ab-plane. For completeness, we note that if the direction of the external magnetic field *h* is reversed, the direction of the total polarization will also reverse (it will be in the direction of $P_{2}$), while the analysis presented above will remain valid, i.e., the qualitative picture of the dependence of P on *h* will not change.

It should be noted that we have calculated the two sublattice magnetizations and polarizations of the material. Moreover, there is a discrepancy in both experimental and theoretical data regarding whether the substance is antiferromagnetic and antiferroelectric. With the help of our model, we show that the compound is antiferromagnetic and antiferroelectric. Total polarization and magnetization can occur only when an external electric and/or magnetic field is applied, which is in disagreement with the reported results of Srivastava et al. [13] but in agreement with those of Deng et al. [14], Khanh et al. [12], and Fang et al. [10].

### 3.3. Temperature and Magnetic Field Dependence of the Dielectric Constant in CNO

**Figure 7:**
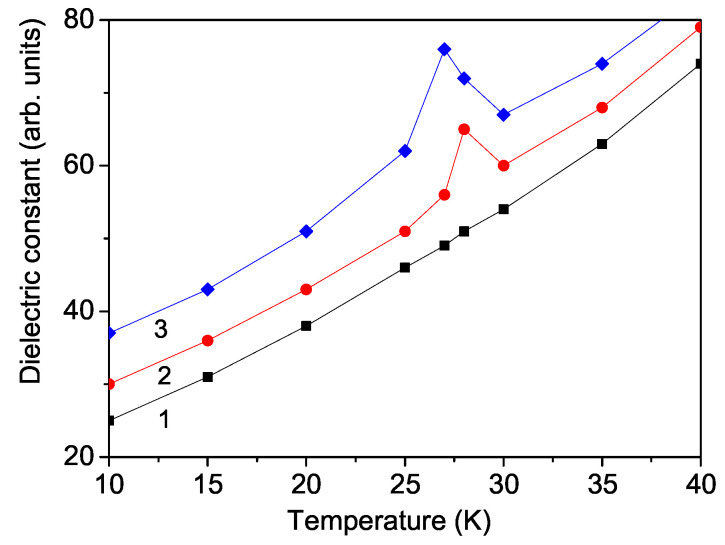
(Color online) Theoretically obtained temperature dependence of the real part of the dielectric constant ϵ in CNO for different external magnetic fields *h*: (1) 0, (2) 2, and (3) 8 kOe.

Finally, we will study the temperature and magnetic field dependence of the real part of the dielectric constant ϵ(T,h) in CNO. It is calculated from Equations (9) and (10). The results are shown in Figure 7. It can be seen that without a magnetic field *h*, there is no peak around the Neel temperature $T_{N}$ in the curve for ϵ(T) (Figure 7, curve 1). The dielectric constant ϵ enhances with increasing temperature *T*. Contrary to us, Yadav et al. [23] reported from temperature-dependent dielectric measurements a dielectric anomaly in CNO at $T_{N}$ even in the absence of an external magnetic field, as well as from Raman studies the presence of additive spin–phonon coupling. The last interaction could be considered in a future paper investigating the phonon energy and damping in CNO.

Applying an external magnetic field *h*, there appears a peak at the Neel temperature $T_{N}$≈ 28 K (see Figure 7, curve 2). Enhancing the magnetic field *h*, the peak increases and shifts to lower temperature values. A similar anomaly in the dielectric constant is also observed by Fang et al. [10]. It can be interpreted as a sudden change in electric polarization induced by spin rotation upon the application of an external magnetic field in the ab-plane. $T_{N}$ decreases with increasing *h*, i.e., the magnetic field tends to suppress the antiferromagnetic transition. Our results are in agreement with those reported by Mufti et al. [6] in ${\mathrm{MnTiO}}_{3}$, by Lee et al. [25] in ${\mathrm{Co}}_{4}{\mathrm{Ta}}_{2}O_{9}$, and by Zhang et al. [26] in ${\mathrm{Fe}}_{4}{\mathrm{Nb}}_{2}O_{9}$.

Let us emphasize that we obtained a similar behavior for the magnetic field dependence of the polarization and dielectric constant in the antiferroelectric cuprate compound ${\mathrm{NaCu}}_{2}O_{2}$ [27].

**Figure 8:**
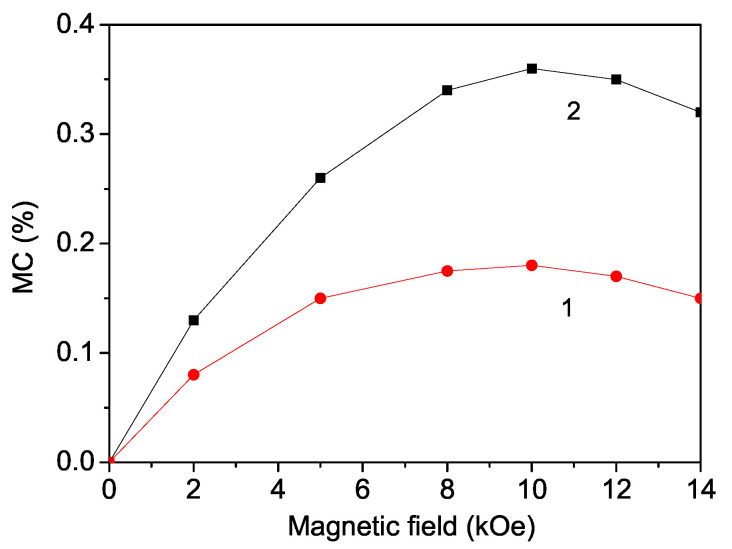
(Color online) Theoretically obtained magnetic field dependence of the magneto-dielectric coefficient MC (%) for different temperatures *T*: (1) 10 and (2) 25 K.

From Equation (11), we have calculated the magneto-dielectric coefficient MD(%). The results for the magnetic field dependence of the MD for two different temperatures, *T* = 20 and 35 K, are presented in Figure 8. It can be seen that the MD firstly increases and then begins to decrease with enhancing the magnetic field *h*. This increase is stronger for higher temperatures *T* (see Figure 8, curve 2). The observed behavior of the magnetic field dependence of the magneto-dielectric coefficient MD(*h*) is in analogy with that of the magnetic field dependence of the polarization P(h) in CNO.

## 4. Conclusions

In conclusion, using, for the first time, Green’s function theory and a microscopic model, the multiferroic properties of the linear ME compound CNO are studied theoretically. Moreover, we tried to clarify the discrepancies in the discussion in the literature of the electric and dielectric behavior of CNO with and without external magnetic fields. Is it an antiferromagnetic and antiferroelectric compound? We have observed that there is no spontaneous electric polarization *P* without an external magnetic field *h*. However, magnetic-field-induced polarization is observed by applying an external magnetic field *h*, as is supported by our schematic representations of the two sublattice polarizations without and with an external magnetic field. The induced polarization *P* increases firstly and then begins to decrease with the increase in *h*. This confirms the existence of strong ME coupling in CNO. The real part of the dielectric constant ϵ without an external magnetic field *h* does not show a peak at the antiferromagnetic phase transition temperature $T_{N}$. But applying *h*, there appears a peak around the Neel temperature $T_{N}$, which increases with raising *h* and then shifts to lower temperatures. The magneto-dielectric coefficient MD(%) increases with enhancing the magnetic field *h* and then decreases for higher *h* values. Moreover, the magnetization *M* increases with an increasing external electric field *E* below the Neel temperature $T_{N}$. $T_{N}$ decreases slightly with increasing electric field *E*. The observed results are in good qualitative coincidence with the existing experimental data. Let us emphasize that further experimental and theoretical works are needed to study the magnetization and polarization of the antiferromagnetic, antiferroelectric linear ME CNO compound.

## Data Availability

Data sharing not applicable to this article as no data sets were generated or analysed during the current study.

## References

[1] Fiebig M. (2005). Revival of the magnetoelectric effect. J. Phys. D.

[2] Mostovoy M. (2024). Multiferroics: Different routes to magnetoelectric coupling. npj Spintron..

[3] Khomskii D.I. (2006). Multiferroics: Different ways to combine magnetism and ferroelectricity. J. Magn. Magn. Mater..

[4] Van Aken B.B., Palstra T.T.M., Filippetti A., Spaldin N.A. (2004). The origin of ferroelectricity in magnetoelectric YMnO_3_. Nat. Mater..

[5] Kimura T., Goto T., Shintani H., Ishizaka K., Arima T., Tokura Y. (2003). Magnetic control of ferroelectric polarization. Nature.

[6] Mufti N., Blake G.R., Mostovoy M., Riyadi S., Nugroho A.A., Palstra T.T.M. (2011). Magnetoelectric coupling in MnTiO_3_. Phys. Rev. B.

[7] Hwang J., Choi E.S., Zhou H.D., Lu J., Schlottmann P. (2012). Magneto-electric effect in NdCrTiO_5_. Phys. Rev. B.

[8] Fischer E., Gorodetsky G., Hornreich R.M. (1972). A new family of magnetoelectric materials: A_2_M_4_O_9_ (A = Ta, Nb; M = Mn, Co). Solid State Commun..

[9] Kolodiazhnyi T., Sakurai H., Vittayakorn N. (2011). Spin-flop driven magneto-dielectric effect in Co_4_Nb_2_O_9_. Appl. Phys. Lett..

[10] Fang Y., Song Y.Q., Zhou W.P., Zhao R., Tang R.J., Yang H., Lv L.Y., Yang S.G., Wang D.H., Du Y.W. (2014). Large magnetoelectric coupling in Co4Nb2O9. Sci. Rep..

[11] Bertaut E.F., Corliss L., Forrat F., Aleonard R., Pauthenet R. (1961). Etude de niobates et tantalates de metaux de transition bivalents. J. Phys. Chem. Solids.

[12] Khanh N.D., Abe N., Sagayama H., Nakao A., Hanashima T., Kiyanagi R., Tokunaga Y., Arima T. (2016). Magnetoelectric coupling in the honeycomb antiferromagnet Co_4_Nb_2_O_9_. Phys. Rev. B.

[13] Srivastava P., Chaudhary S., Maurya V., Saha J., Kaushik S., Siruguri V., Patnaik S. (2018). Magnetic structure driven ferroelectricity and large magnetoelectric coupling in antiferromagnet Co_4_Nb_2_O_9_. Solid State Commun..

[14] Deng G., Cao Y., Ren W., Cao S., Studer A.J., Gauthier N., Kenzelmann M., Davidson G., Rule K.C., Gardner J.S. (2018). Spin Dynamics and Magnetoelectric Coupling Mechanism of Co_4_Nb_2_O_9_. Phys. Rev. B.

[15] Yanagi Y., Hayami S., Kusunose H. (2018). Manipulating the magnetoelectric effect: Essence learned from Co_4_Nb_2_O_9_. Phys. Rev. B.

[16] Matsumoto M., Koga M. (2019). Symmetry analysis of magnetoelectric effects in honeycomb antiferromagnet Co_4_Nb_2_O_9_. J. Phys. Soc. Jpn..

[17] Solovyev I.V., Kolodiazhnyi T.V. (2016). Origin of magnetoelectric effect in Co_4_Nb_2_O_9_ and Co_4_Ta_2_O_9_: The lessons learned from the comparison of first-principles-based theoretical models and experimental data. Phys. Rev. B.

[18] Tserkovnikov Y.A. (1971). Decoupling of chains of equations for two-time Green’s functions. Theor. Math. Phys..

[19] Katsura H., Nagaosa N., Balatsky A.V. (2005). Spin current and magnetoelectric effect in noncollinear magnets. Phys. Rev. Lett..

[20] Yamasaki Y., Miyasaka S., Kaneko Y., He J.-P., Arima T., Tokura Y. (2006). Magnetic reversal of the ferroelectric polarization in a multiferroic spinel oxide. Phys. Rev. Lett..

[21] Vaks V.G. (1973). Introduction to the Microscopic Theory of Ferroelectrics.

[22] Cao Y., Deng G., Beran P., Feng Z., Kang B., Zhang J., Guiblin N., Dkhil M., Ren W., Cao S. (2017). Nonlinear magnetoelectric efect in paraelectric state of Co_4_Nb_2_O_9_ single crystal. Sci. Rep..

[23] Yadav S., Chandra M., Rawat R., Khandelwal A., Chandra J.S.S., Choudhary R.J., Sathe V. (2022). Temperature-Dependent Structural, Dielectric, and Raman Spectroscopy Studies on Magnetoelectric Co_4_Nb_2_O_9_. J. Phys. Chem. C.

[24] Malashevich A., Coh S., Souza I., Vanderbilt D. (2012). Full magnetoelectric response of Cr_2_O_3_ from first principles. Phys. Rev. B.

[25] Lee N., Oh D.G., Choi S., Moon J.Y., Kim J.H., Shin H.J., Son K., Nuss J., Kiryukhin V., Choi Y.J. (2020). Highly nonlinear magnetoelectric effect in buckled-honeycomb antiferromagnetic Co_4_Ta_2_O_9_. Sci. Rep..

[26] Zhang J., Su N., Mi X., Pi M., Zhou H., Cheng J., Chai Y. (2021). Probing magnetic symmetry in antiferromagnetic Fe_4_Nb_2_O_9_ single crystals by linear magnetoelectric tensor. Phys. Rev. B.

[27] Apostolova I.N., Apostolov A.T., Wesselinowa J.M. (2021). Multiferroic and phonon properties at the phase transition of S = 1/2 chain cuprates NaCu_2_O_2_. Comparison with LiCu_2_O_2_. Phase Transit..

